# Age-dependent and regional heterogeneity in the long-chain base of A-series gangliosides observed in the rat brain using MALDI Imaging

**DOI:** 10.1038/s41598-017-16389-z

**Published:** 2017-11-23

**Authors:** Sarah Caughlin, Shikhar Maheshwari, Nina Weishaupt, Ken K-C Yeung, David Floyd Cechetto, Shawn Narain Whitehead

**Affiliations:** 1 Vulnerable Brain Laboratory, Department of Anatomy and Cell Biology, Schulich School of Medicine and Dentistry, University of Western Ontario, London, ON N6A 5C1 Canada; 2 Department of Chemistry, Department of Biochemistry, Schulich School of Medicine and Dentistry, University of Western Ontario, London, ON N6A 5C1 Canada

## Abstract

Alterations in the long chain base of the sphingosine moiety of gangliosides have been shown to play a role in neurodevelopment and neurodegeneration. Indeed, the accumulation of d20:1 sphingosine has been referred to as a metabolic marker of aging in the brain, however, this remains to be shown in simple gangliosides GM2 and GM3. In this study, Matrix-assisted laser desorption/ionization Imaging Mass Spectrometry (MALDI IMS) was used to examine the neuroanatomical distribution of A-series gangliosides with either 18 or 20 carbon sphingosine chains (d18:1 or d20:1) in Fisher 344 rats across the lifespan. The ratio of d20:1/d18:1 species was determined across 11 regions of interest in the brain. Interestingly, a decrease in the d20:1/d18:1 ratio for GM2 and GM3 was observed during early development with the exception of the peri-ventricular corpus callosum, where an age-dependent increase was observed for ganglioside GM3. An age-dependent increase in d20:1 species was confirmed for complex gangliosides GM1 and GD1 with the most significant increase during early development and a high degree of anatomical heterogeneity during aging. The unique neuroanatomically-specific responses of d20:1 ganglioside abundance may lead to a better understanding of regional vulnerability to damage in the aging brain.

## Introduction

Gangliosides are a class of glycosphingolipids that are found throughout all cells of the body, with certain species enriched in the central nervous system (CNS). They are part of a large family of lipid species that form an important structural and functional component of lipid rafts, a functional domain of the cell membrane enriched in phospholipids, cholesterol, and gangliosides in which protein-lipid interactions occur leading to signal transduction^1,2^. Within the CNS, each ganglioside appears to have unique effects on signal transduction. For example, ganglioside GM1 has been shown to enhance neuroprotection through modulation of neurotrophin release^3^ and ion transport^4^, while accumulation of ganglioside GM3 has been shown to lead to apoptotic cell death in astrocytes^5^ and neurons^6^. Moreover, perturbations in the homeostatic distribution of gangliosides has been observed in rodent models of brain injury such as middle cerebral arterial occlusion (MCAO) stroke^7^, co-morbid stroke and amyloid beta toxicity^8^, and traumatic brain injury^9^, as well as in preclinical models and human patients with neurodegenerative diseases^10–14^. Thus, there has been increasing interest in the maintenance/enhancement of ganglioside homeostasis as a treatment for neurodegenerative conditions.

**Figure 1:**
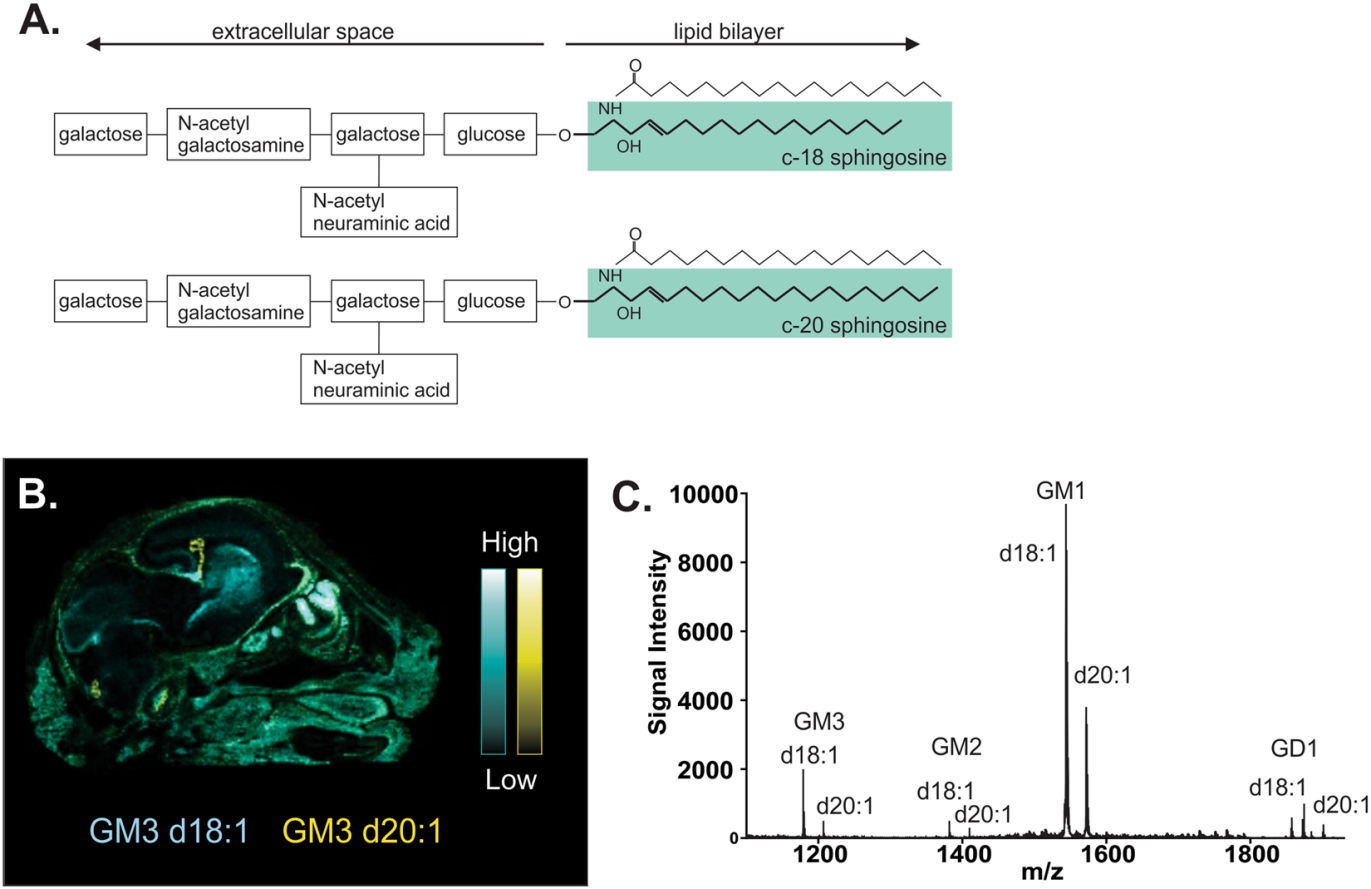
Ganglioside structure and detection using MALDI IMS. Chemical structure and MALDI IMS of d18:1 and d20:1 gangliosides. (**A**) Gangliosides are composed of both a hydrophilic domain which extends into the extracellular space, and hydrophobic ceramide anchor (highlighted) which is embedded in the cell membrane. The hydrophillic portion contains an oligosaccharide chain (Glc, Gal, GalNac) and sialic acid residues (NeuAc) which determines the type of ganglioside (i.e. GM1, GM2, GM3). The hydrophobic portion of the molecule is made up of a fatty acid, usually stearic acid, and a sphingosine LCB tail with varying numbers of carbons. (**B**) Representative MALDI IMS molecular image depicting anatomical distribution of d18:1 (blues), and d20:1 (yellow) GM3 species across a sagittal section of a P0 Fisher 344 rat head. (**C**) Representative mass spectrum using DAN matrix in negative reflection mode depicting the mass of A-series gangliosides (and corresponding m/z values) analyzed in the current study. From left to right: GM3 d18:1 (1179 Da), GM3 d20:1 (1207 Da), GM2 d18:1 (1382 Da), GM2 d20:1 (1409 Da), GM1 d18:1 (1543 Da), GM1 d20:1 (1572 Da), GD1a[K^+^] d18:1 (1872 Da) and GD1a[k^+^] d20:1 (1901 Da).

Gangliosides are structurally composed of a hydrophilic domain, containing sialic acid residues attached to an oligosaccharide chain, along with a hydrophobic domain made up of a ceramide complex (Fig. 1A). Ceramide is composed of a fatty acid attached to a sphingosine long-chain base (LCB). Gangliosides can be differentiated from each other based on the length of their oligosaccharide chain and the number of sialic acid residues in their hydrophilic domain, which determines the type of ganglioside as described by their designation (e.g. ganglio-monosialo 3 or GM3). Gangliosides can also be differentiated by the type of fatty acid and number of carbons present within their ceramide domain. The 18 carbon sphingosine, chemically denoted as d18:1, is the predominant species in brain gangliosides, with 20 carbon species (d20:1) being present in lower, but variable quantities^15^.

Alterations in the LCB have been linked to neurodevelopment and also implicated as a potential mechanism in the development of neurodegeneration^8,16^. Structurally, the additional carbons present on the ceramide moiety lead to an increase in volume of the hydrophobic portion of the molecule. This alters the organization of the membrane and its fluid properties^17^. Changes in the organization of the membrane has consequences for the ability of gangliosides in lipid-rich domains to interact with the external environment and carry out their function as modulators of cell signalling^18^. Therefore, the presence of d18:1 or d20:1 species in the membrane may alter the effectiveness of signal transduction at the cell surface. The d20:1 species have also been referred to as a metabolic marker of aging^19^ as these species have consistently been observed to increase with age in murine^15,19–22^ and human brains^23^. However, the literature on this topic has focused almost exclusively on major complex gangliosides GM1, GD1, GT1, and GQ1, with minimal neuroanatomically-specific information, and has not described how the LCB of minor, simple gangliosides shift during aging. This information may be crucial as there is increasing evidence pointing to the potential role of simple ganglioside, such as GM3 and GM2, in the development and pathogenesis of neurodegenerative diseases and injuries^7–9^, however, the role of the ceramide moiety remains unclear due to technical challenges in the detection of these low abundance species.

Previous investigations have used Matrix Assisted Laser Desorption Ionization Imaging Mass Spectrometry (MALDI-IMS) to visualize d18:1 and d20:1 species of ganglioside GM1^7,8,20,24^. This technique has the advantage of simultaneously detecting multiple species of gangliosides (and other molecules) within the same sample based on their abundance and neuroanatomical location. This tool is also a powerful technique to differentiate carbon numbers within ganglioside LCB, which cannot be done using antibody labelling. A study describing the expression of d18:1 and d20:1 species of all A-series gangliosides during the aging process has not yet been done. Therefore, the following study provides the first detailed examination of age-dependent changes in the LCB of both simple and complex A-series gangliosides across a large number brain regions in wildtype (Wt) and APP21 transgenic (Tg) Fisher 344 rats that contain the human mutations to the amyloid precursor protein^24,25^. We hypothesized that there would be significant regional differences in the ratio of d20:1 species relative to d18:1 across the brain throughout aging and that this would be enhanced in the Tg APP21 rat. To achieve this, we used MALDI IMS (Fig. 1B,C), a sophisticated analytical imaging technology capable of detecting and visualizing gangliosides based on their ionic mass, allowing for accurate neuroanatomically-localized detection of d18:1 and d20:1 species across intact tissue sections.

## Materials and Methods

### Animal Model

All procedures involving live animals were in accordance with the guidelines of the Canadian Council on Animal Care and approved by the University of Western Ontario Animal Use Committee (Protocol 2014–2016). Wt and Tg APP21 Fisher 344 rats were kindly provided by Dr. Yukel Agca (University of Missouri) and bred in-house. Tg APP21 rats contained both human Swedish and Indiana mutations for the amyloid precursor protein gene. Because there were no significant transgene effects at each individual time point, the data for both Wt and Tg APP21 rats was pooled for all analysis for a final n value of 10 per group. Rats were aged to 4 different time points to be as representative as possible of the full lifespan, leading to a total of 40 rats included in the study. Rats were sacrificed via fresh-frozen extraction (described elsewhere) at either P0 (newborn), 3 months (m, young rats), 12 m (middle aged), or 20 m (old). Brains were stored at −80 °C until processed for MALDI IMS.

### MALDI IMS

Brain tissue (or whole heads for P0 rats) were sectioned on a cryostat (Thermo-Fisher Scientific CryoStar NX50) at a thickness of 10 μm and thaw mounted onto electrically conductive Indium-Tin-Oxide (ITO) slides (Hudson Surface Technology Inc., Old Tappan, NY, USA). The slides were then coated in a thin layer of 1,5-Diaminonapthalene (DAN, Sigma-Aldrich, Oakville, ON, Canada) matrix via sublimation and incubated at −20 °C overnight. After a 10 min desiccation period, a 5 peptide calibration standard was applied (Sciex, Farmingham, MA. USA) and allowed to dry. An image of the plate was scanned for reference in the instrument and the instrument calibrated using the spotted standards to a mass tolerance of 50 ppm. Images were acquired using a Sciex MALDI 5800 TOF/TOF instrument in negative ion reflectron mode. MS images were acquired at a 70 μm raster with 20 shots/spectrum. This protocol has been previously described in detail^26^.

### Data Analysis

The regions of interest (ROIs) were confirmed using the rat brain atlas by Paxinos & Watson (1998). A total of 11 ROIs were quantified across the brain and were grouped into discrete anatomical/functional regions for quantitation. These clustered groups include: sub-cortical structures (striatum, lateral septal nucleus, and substantia nigra), the cortex (superficial, intermediate, and deep layers of the cortex), the hippocampus (CA1, dentate gyrus molecular layer, dentate gyrus granular layer), and white matter (anterior corpus callosum and peri-ventricular corpus callosum).

MALDI IMS images were analyzed using TissueView software (Version 2, Sciex). A total of 4 tissue sections per brain were used to isolate discrete anatomical regions in order to quantify the 11 regions of interest, for a total of 160 MALDI MS images (40 rats × 4 sections) and 440 total datasets (40 rats × 11 ROIs). Peaks corresponding to major A-series gangliosides were isolated and identified, as confirmed by previous studies^9,20,27,28^ and the Lipidmaps database (www.lipidmaps.org). For quantification of MALDI-IMS data, the area under the curve (AUC) of the three largest isotopic peaks was determined and a ratio was obtain between the AUC of each species and total AUC. This corrected value was used to perform a ratio of the d20:1 species of each A-series ganglioside to the d18:1 species. This ratio value was used to make between group comparisons so as to reduce error produced by variability in sample preparation between images as previously published^26^. For GD1, the more abundant K^+^ adduct peaks were used. Statistical analysis was performed using either a student’s t-test or a two-way ANOVA, followed by a Tukey’s post-hoc test.

## Results

### LCB Genotype differences between Wt and Tg APP21 rats were restricted to GM3 within the peri-ventricular corpus callosum

No significant differences in regional abundance across age were observed between Wt and Tg APP21 rats with the exception of ganglioside GM3 in the peri-ventricular corpus callosum where the fold increase of d20:1/d18:1 between P0 and 20 m was found to be significantly higher in Tg APP21 rats than their Wt counterparts (Supplemental Fig. 1). Given the lack of genotype specific differences in d20:1/d18:1 ratios at each individual time point, all future analysis used pooled data from both Wt and Tg APP21 rats.

### Significant anatomical heterogeneity throughout the rat life span within subcortical and basal ganglia structures for gangliosides GM1 and GM3

**Figure 2:**
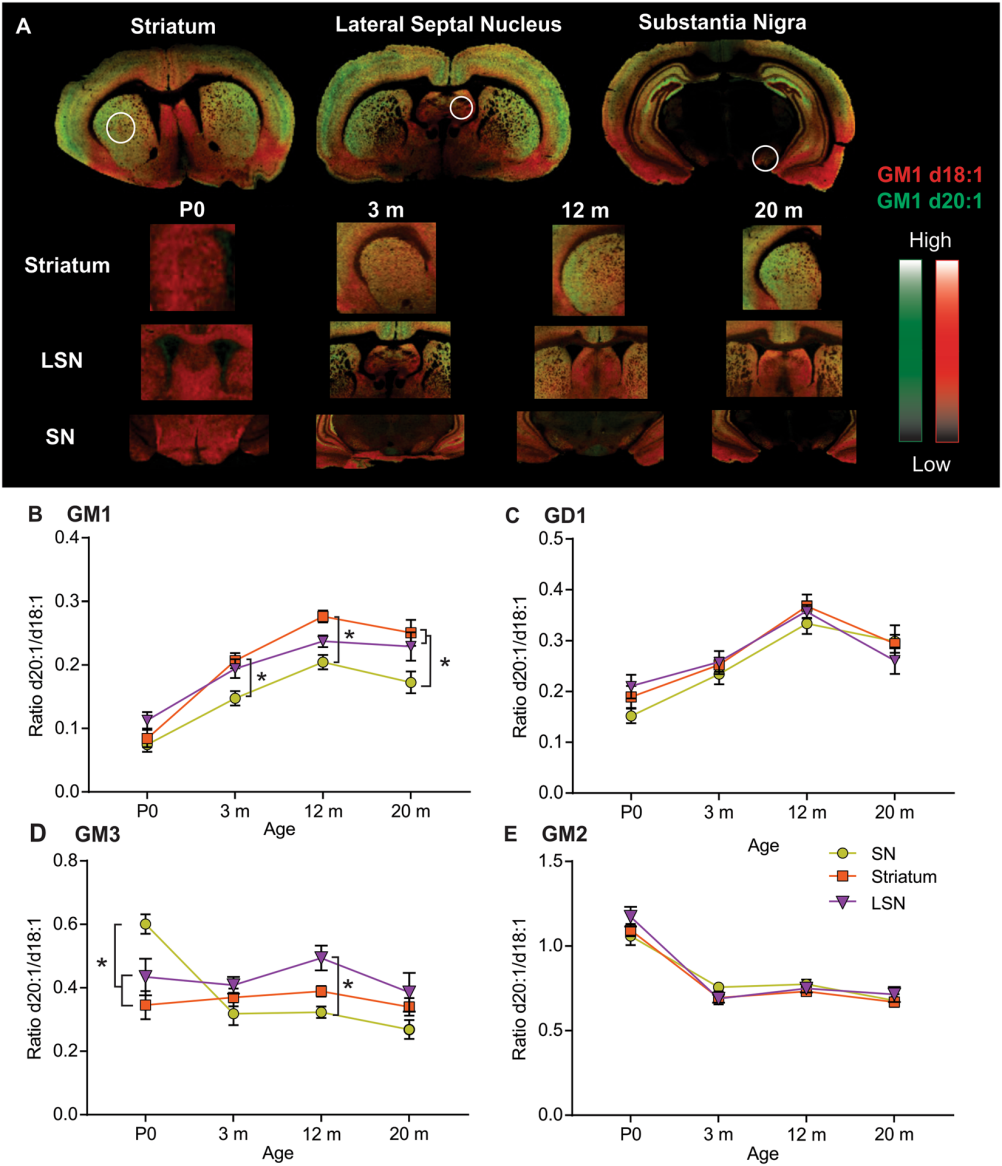
Significant regional heterogeneity in ganglioside LCB ratio among sub-cortical structures. Visualization of regional and age-dependent shifts in a-series gangliosides in sub-cortical rat brain structures. (**A**) Representative MALDI IMS overlay images showing the anatomical distribution of GM1 d18:1 (red) and d20:1 (green). (**B–E**) Quantification of the d20:1/d18:1 ratio at each time point showing the significant regional differences for each A-series ganglioside, GM1 (**B**), GD1 (**C**), GM3 (**D**), and GM2 (**E**). The d20:1/d18:1 LCB ratio increased in an age-dependent manner for complex gangliosides GM1 and GD1 up to 12 m then plateaued up to 20 m (GM1) or decreased slightly (GD1). Significant anatomical hetereogeneity in the LCB ratio was observed from simple ganglioside GM3 among sub-cortical structures, with a decrease during early development in the SN, and a stable ratio in the striatum and LSN throughout the lifespan. Data represented as group Means ± SEM, *indicates statistical significance, p < 0.05, via two-way ANOVA, Tukey multiple comparisons test, n = 10 for each time point.

Sub-cortical structures are susceptible to age related changes associated with Alzheimer’s disease (basal forebrain) Parkinson’s disease (substantia nigra) and Huntington’s disease (striatum). We measured the ratio between d20:1 and d18:1 species of gangliosides within the lateral septal nucleus (LSN) of the basal forebrain, substantia nigra (SN), and striatum of rats from P0 to 20 m of age (Fig. 2A). There were no regional differences in GM1 d20:1/d18:1 ratios in P0 rats. At 3 m, the d20:1/d18:1 ratio was significantly lower in the SN (0.15) than the striatum (0.21), and remained the lowest of the three regions up to 20 m (Fig. 2B). At 12 m, the ratio of GM1 d20:1/d18:1 species was significantly higher in the striatum (0.28) than the SN (0.20), with the LSN between the two (0.24), however, by 20 m, the regional differences shifted back to a similar pattern as that of 3 m, with both the striatum (0.25) and LSN (0.23) demonstrating significantly higher GM1 d20:1/d18:1 ratios than the SN (0.17). When measuring differences in d18:1/d20:1 ratios for GD1, no regional differences were observed at any time point in the basal ganglia (Fig. 2C). The GD1 d20:1/d18:1 ratio increased from P0 to 12 m in all subcortical structures then dropped slightly by 20 m.

With respect to simple ganglioside GM3 during aging, the d20:1/d18:1 ratio increased slightly from P0 (0.43, 0.34) to 12 m (0.49, 0.39) within the LSN and striatum respectively. The d20:1/d18:1 GM3 ratio within the SN decreased significantly from 0.6 in P0 rats to 0.32 at 3 m, which then continued to decrease slightly up to 20 m (0.27) (Fig. 2D). These divergent patterns for the GM3 LCB ratio during aging are indicative of significant regional differences within subcortical structures. Indeed, at P0, a regional difference was observed with significantly higher d20:1 content observed in the SN (0.6) over the striatum (0.35) and LSN (0.43). This pattern shifted at 12 m where the SN d20:1/d18:1 ratio decreased to 0.32 while the LSN ratio increased to 0.49, leading to a significant difference between these two brain regions. These regional differences disappeared at 20 m as the d20:1/d18:1 ratio dropped in the LSN (0.38) (Fig. 2D). significant at 20 m. There were no regional differences observed in the GM2 species (Fig. 2E), however, all brain regions showed a significant decrease in the d20:1/d18:1 ratio between P0 and 3 m which then remained stable up to 20 m.

### Cortical layers show regional heterogeneity in ganglioside of LCB length

**Figure 3:**
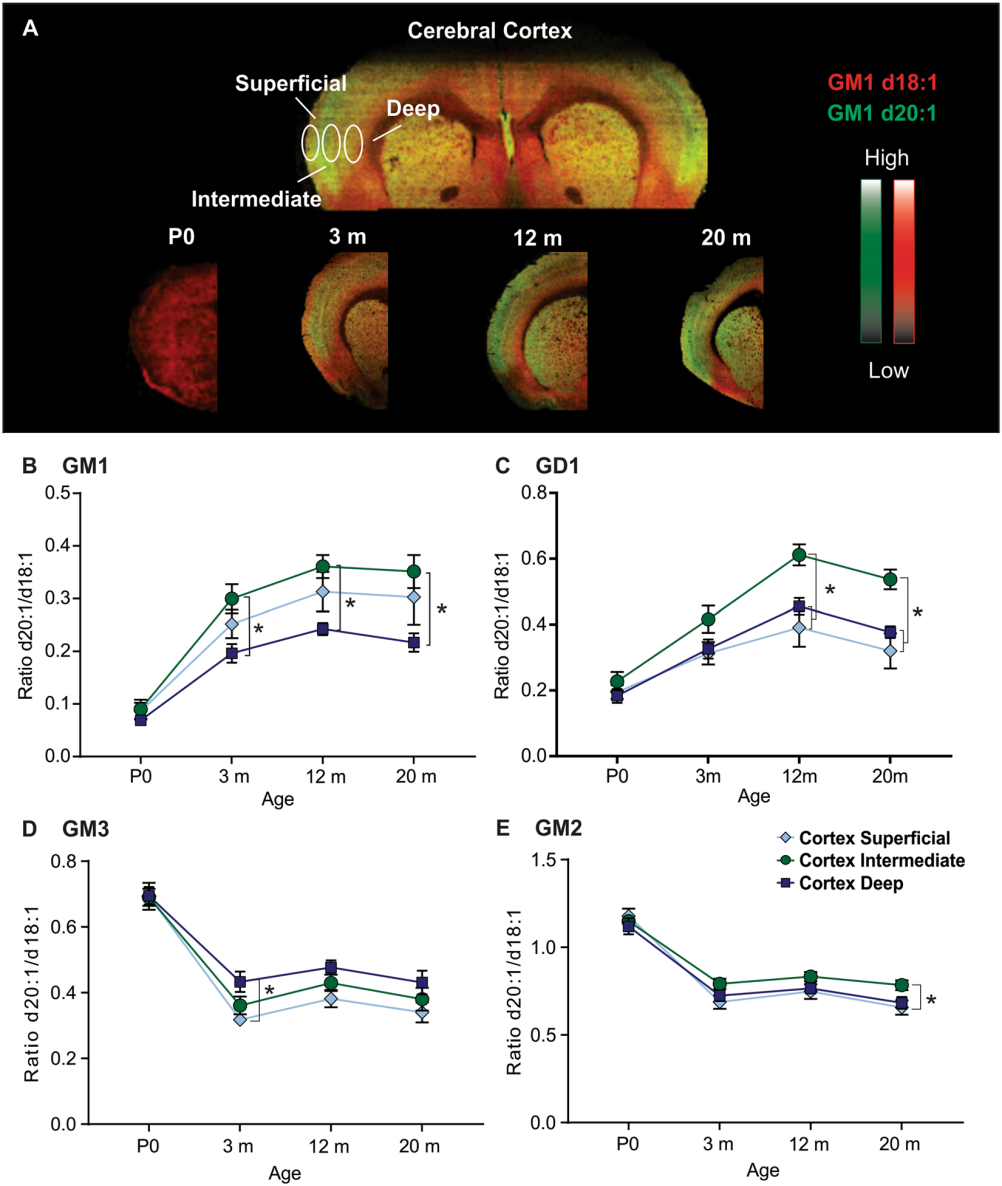
LCB ratio highest in superficial layers of the cortex and shows opposite pattern during early development among complex and simple gangliosides. Visualization of regional and age-dependent shifts in A-series gangliosides in the cerebral cortex of the rat. (**A**) Representative MALDI IMS overlay images showing the anatomical distribution of GM1 d18:1 (red) and d20:1 (green) across tissue along with the anatomical location of the ROIs in the CA1 region, dentate gyrus molecular layer (DG mol), and dentate gyrus granular layer (DG gr). (**B–E**) Quantification of the d20:1/d18:1 ratio at each time point showing the significant regional differences across the lifespan for each A-series ganglioside, GM1 (**B**), GD1 (**C**), GM3 (**D**), and GM2 (**E**). The ratio of d20:1/d18:1 species increased in an age-dependent manner in complex gangliosides GM1 and GD1 up to 12 m of age, with the highest ratio observed in the intermediate layers of the cortex. The ratio then plateaued or decreased slightly at 20 m. The LCB ratio dropped between P0 and 3 m in simple gangliosides GM3 and GM2. Data represented as group Means ± SEM, *indicates statistical significance, p < 0.05, via two-way ANOVA, Tukey multiple comparisons test, n = 10 for each time point.

Regional differences between the superficial, intermediate, and deep layers of the cerebral cortex have previously been reported^24^. The present study used similar cortical ROIs (Fig. 3A) to evaluate alterations in the LCB length during aging in the rat. When measuring the LCB length of GM1, a similar d20:1/d18:1 ratio of 0.07–0.09 was observed in all three layers of the cortex of P0 rats (Fig. 3B). All three layers demonstrated variable but increased d20:1/d18:1 GM1 during aging with a plateau observed at 12 m. Interestingly, the intermediate layer demonstrated the highest ratio of d20:1/d18:1 expression for GM1 which became apparent at 3 m (intermediate – 0.30, superior – 0.25, deep – 0.20) and continued to be significantly higher than the deep layers up to 20 m. A similar pattern was observed for LCB length of GD1 whereby P0 rats had similar ratios of d20:1/d18:1 throughout all layers of the cortex followed by an increase in the ratio of d20:1/d18:1 at 3 m and a plateau at 12 m (Fig. 3C). Like GM1, GD1 in the intermediate layer of the cortex also demonstrated the greatest increase in the LCB ratio which was most apparent at 12 m (intermediate – 0.61, superficial – 0.39, deep – 0.45) and continued to be significantly higher than the other cortical layers up to 20 m.

The aging abundance profile of simple gangliosides GM3 and GM2 LCBs was the opposite of their complex ganglioside counterparts whereby the d20:1/d18:1 ratio was highest at P0, and then decreased during early development, plateauing at 3 m (Fig. 3D,E). The deep layers of the cortex showed a significantly higher d20:1/d18:1 ratio at 3 m (0.43) than the superficial layers (0.32) (Fig. 3D). GM2 demonstrated regional differences only at 20 m (Fig. 3E), with the intermediate layers of the cortex showing a significantly higher d20:1/d18:1 LCB ratio (0.78) than the superficial layers (0.66).

### Hippocampus: Age-dependent accumulation in d20:1/d18:1 species in the dentate gyrus molecular layer observed in complex gangliosides only

**Figure 4:**
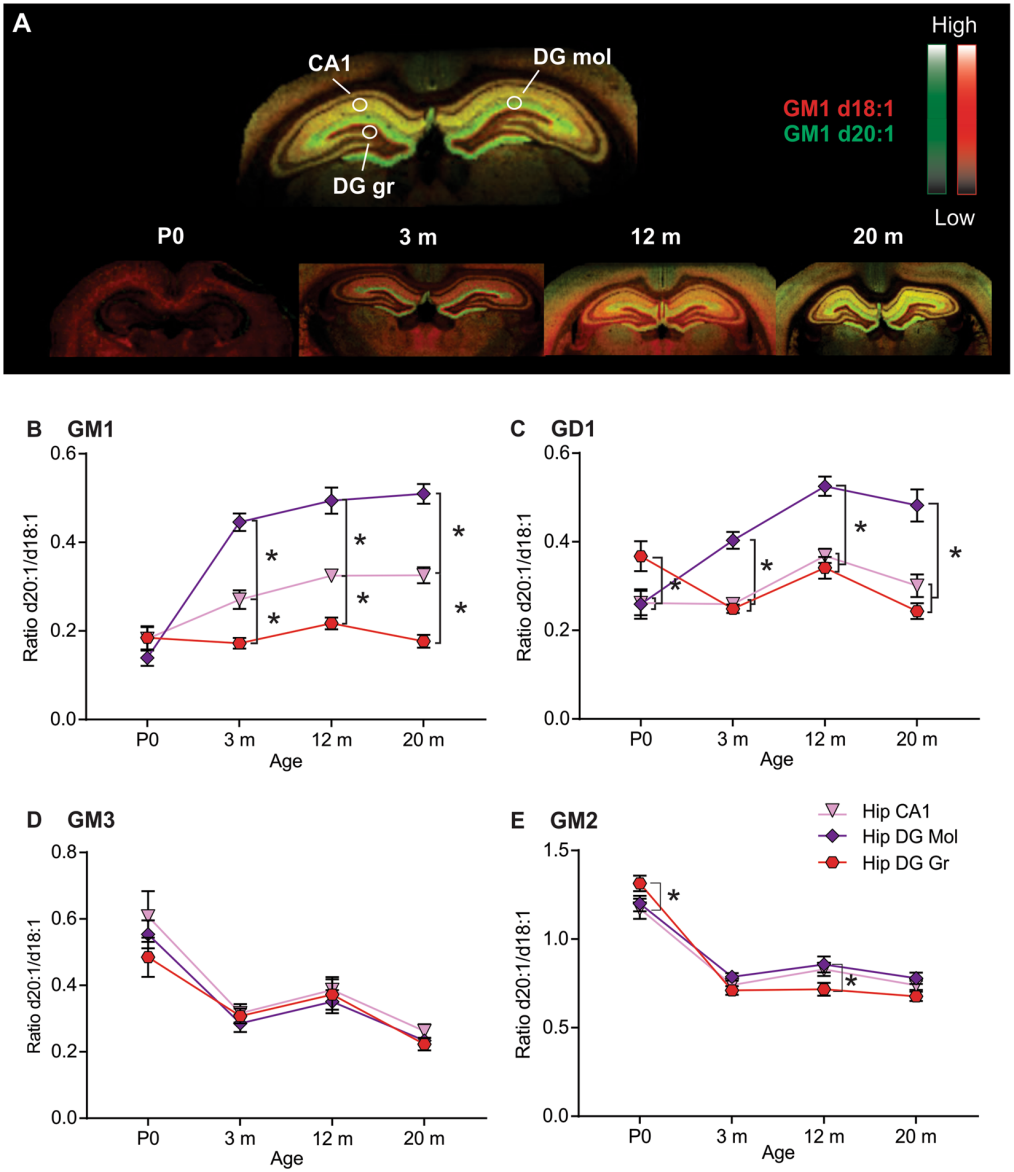
Significant regional heterogeneity in d20:1/d18:1 ratio during aging among hippocampal layers. Visualization of regional and age-dependent shifts in A-series gangliosides in the hippocampus of the rat. (**A**) Representative MALDI IMS overlay images showing the anatomical distribution of GM1 d18:1 (red) and d20:1 (green). (**B–E**) Quantification of the d20:1/d18:1 ratio at each time point showing the significant regional differences across the lifespan for each A-series ganglioside, GM1 (**B**), GD1 (**C**), GM3 (**D**), and GM2 (**E**). Significant anatomical heterogeneity in the age-dependent accumulation of d20:1 species was observed in the various sub-regions of the hippocampus for complex gangliosides GM1 and GD1 while the LCB ratio dropped during early development in simple gangliosides GM3 and GM2. Data represented as group Means ± SEM,* indicates statistical significance, p < 0.05, via two-way ANOVA, Tukey multiple comparisons test, n = 10 for each time point.

Three regions of the hippocampus (Fig. 4A) were selected for analysis based on previous reports of regional differences in the ratio of d20:1/d18:1 for GM1^20,24^. The d20:1/d18:1 ratio of GM1 increased in an age-dependent manner within the CA1 and dentate gyrus (DG) molecular layer (mol) of the hippocampus, however, the ratio remained stable in the granular layer (gr) of the DG throughout the rat lifespan (Fig. 4B). There were no regional differences within the hippocampus at P0, however, pronounced regional differences were observed between all three layers post-natally. The ratio of d20:1/d18:1 GM1 was highest in the DG mol where it increased from 0.14 at P0 to 0.44 at 3 m and continued to increase up to 20 m (0.51). A similar pattern was observed within the CA1 (P0–0.18, 3 m – 0.27, 20 m 0.33). The DG gr showed the lowest d20:1/d18:1 ratio of GM1 within the hippocampus remaining relatively unchanged from P0 (0.18) to 20 m (0.17). As noted in the previous brain regions, the d20:1/d18:1 ratio of GD1 was higher at P0 than for GM1. However, unlike GM1, not all layers of the hippocampus showed age-dependent increases in the ratio of d20:1/d18:1 species. This trend was only observed in the DG mol where the d20:1/d18:1 ratio increased from 0.26 at P0 to 0.4 at 3 m, and continued to increase to 0.52 at 12 m, where it plateaued up to 20 m (Fig. 4C). The DG gr demonstrated an unexpected decrease in the ratio of GD1 d20:1/d18:1 between P0 (0.37) and 3 m (0.25) which then increased at 12 m (0.34) only to drop back down at 20 m (0.24). Equally interestingly, the CA1 region showed no change in the LCB ratio between P0 and 3 m, but increased between 3 m (0.26) and 12 m (0.37), then dropped slightly at 20 m (0.3).

As was observed in the cortex, the ratio of d20:1/d18:1 for simple gangliosides GM2 and GM3 was highest at P0 followed by a large decrease at 3 m where it remained at this level or decreased slightly at 20 m (Fig. 4D,E). There were no regional differences in the GM3 LCB ratio within the hippocampus, however, the d20:1/d18:1 ratio did decreased between P0 and 20 m (CA1 0.61–0.26, DG mol 0.55–0.22, DG gr 0.48–0.22), with the most significant drop occurring in early development. There were modest but statistically significant differences in the GM2 d20:1/d18:1 ratio at P0 (Fig. 4D; CA1 – 1.17, DG gr – 1.31) and 12 m (DG mol – 0.86, DG gr – 0.72) within the hippocampus.

### Age-dependent increase in LCB ratio observed in the white matter for both complex gangliosides and simple ganglioside GM3

**Figure 5:**
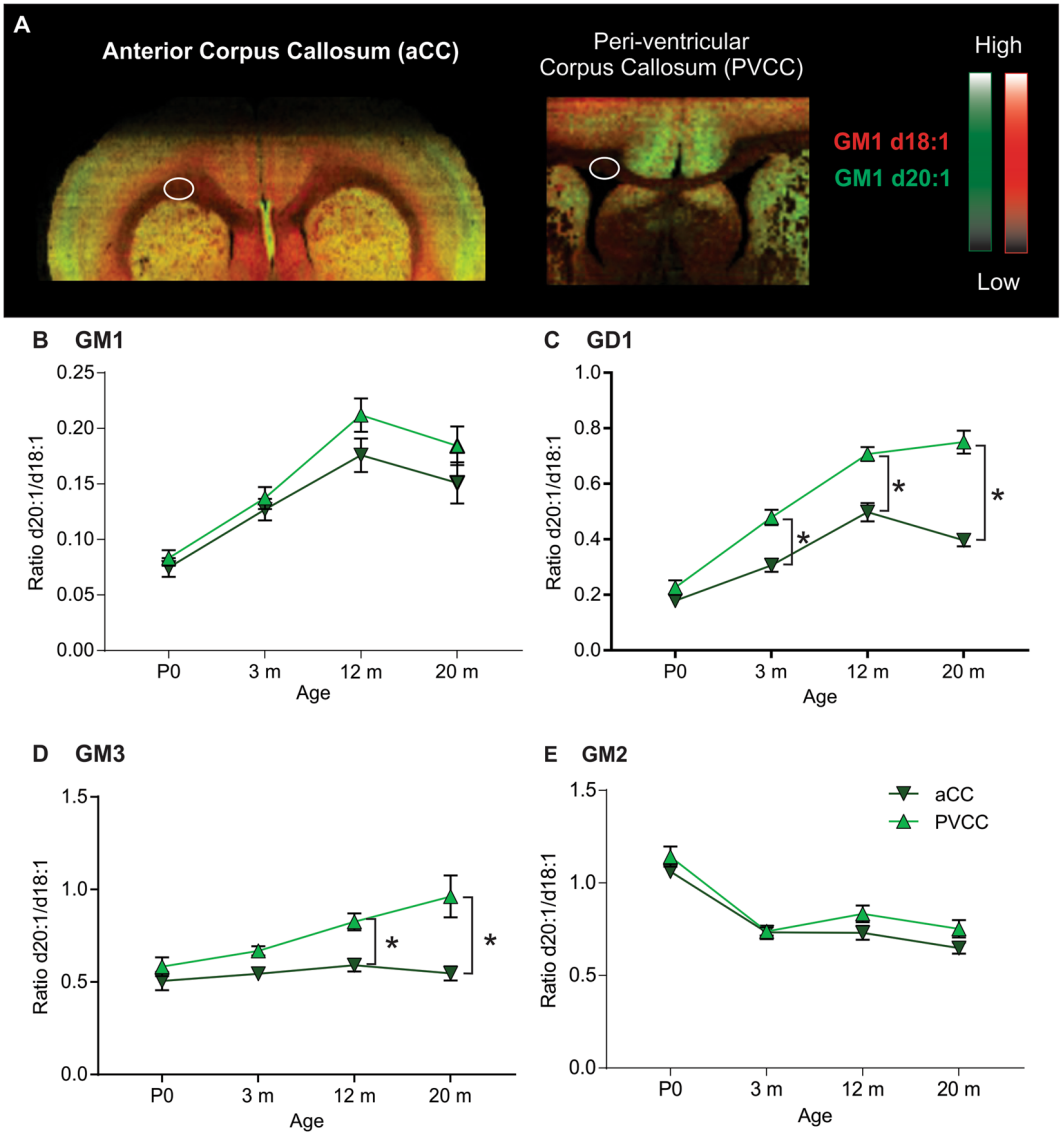
Unique pattern of d20:1 LCB accumulation in the white matter for simple ganglioside GM3 as well as significant differences among white matter regions during aging. Visualization of regional and age-dependent shifts in A-series gangliosides in the white matter of the rat. (**A**) Representative MALDI IMS overlay images showing the anatomical distribution of GM1 d18:1 (red) and d20:1 (green) (**B–E**) Quantification of the d20:1/d18:1 ratio at each time point showing the significant regional differences across the lifespan for each A-series ganglioside, GM1 (**B**), GD1 (**C**), GM3 (**D**), and GM2 (**E**). An age-dependent increase in the d20:1/d18:1 ratio was observed in the PVCC for gangliosides GM1, GD1, and also simple ganglioside GM3. Data represented as group Means ± SEM,*indicates statistical significance, p < 0.05, via two-way ANOVA, Tukey multiple comparisons test, n = 10 for each time point.

Changes in white matter have been linked to several aging related neurodegenerative diseases. We investigated two distinct regions within the corpus callosum that clinically demonstrate differing vulnerability to structural changes in AD patients^29^ (Fig. 5A). The two regions used in our analysis were the anterior corpus callosum (aCC) adjacent to the striatum, and a more caudal sampling from the white matter adjacent to the ventricles, peri-ventricular corpus callosum (PVCC). Interestingly, the PVCC expressed higher ratios of d20:1/d18:1 ganglioside species than the more rostral aCC. Both GM1 and GD1 gangliosides showed an increase in d20:1 species up to 12 m which then plateaued to 20 m (Fig. 5B,C). For GM1 (Fig. 5B), the d20:1/d18:1 ratio increased significantly from P0 (aCC–0.07, PVCC – 0.08) to 3 m (aCC – 0.13, PVCC – 0.14), and continued to increase at 12 m (aCC – 0.18, PVCC – 0.21). A more pronounced age-dependent increase in d20:1 species was observed for ganglioside GD1 in the PVCC. The d20:1/d18:1 ratio increased between each time point (Fig. 5C; P0 – 0.22, 3 m – 0.48, 12 m – 0.7, 20 m – 0.75). For the aCC, a similar pattern was observed to that of the PVCC, however, the d20:1/d18:1 ratio dropped between 12 m (0.5) and 20 m (0.4). Interestingly, regional differences were observed between the two white matter regions for the GD1 LCB ratio which became apparent at 3 m (PVCC – 0.48, aCC – 0.3) and continued at 12 m (PVCC – 0.7, aCC – 0.5) and 20 m (PVCC – 0.75, aCC – 0.4).

The aging shifts of LCBs were completely different between simple gangliosides GM2 and GM3 (Fig. 5D,E). The ratio of d20:1/d18:1 for GM3 increased steadily in the PVCC throughout aging, similar to what was observed for GM1 and GD1 (Fig. 5D; P0 – 0.58, 3 m – 0.67, 12 m – 0.82, 20 m – 0.96) whereas the aCC remained relatively constant (P0 – 0.5, 3 m – 0.54, 12 m – 0.59, 20 m – 0.55) resulting in significant differences between the aCC and PVCC ratios at 12 and 20 m of age. This was the only brain region to show an increase in the GM3 d20:1/d18:1 ratio during aging. Simple ganglioside GM2 (Fig. 5E) showed a similar pattern to that of the other brain regions, with a significant decrease in the d20:1/d18:1 ratio from P0 (aCC–1.06, PVCC–1.14) to 3 m (aCC–0.74, PVCC – 0.74) where the ratio remained unchanged up to 20 m in the PVCC (0.75) and dropped slightly in the aCC (0.65).

### Overall Age Effects

**Figure 6:**
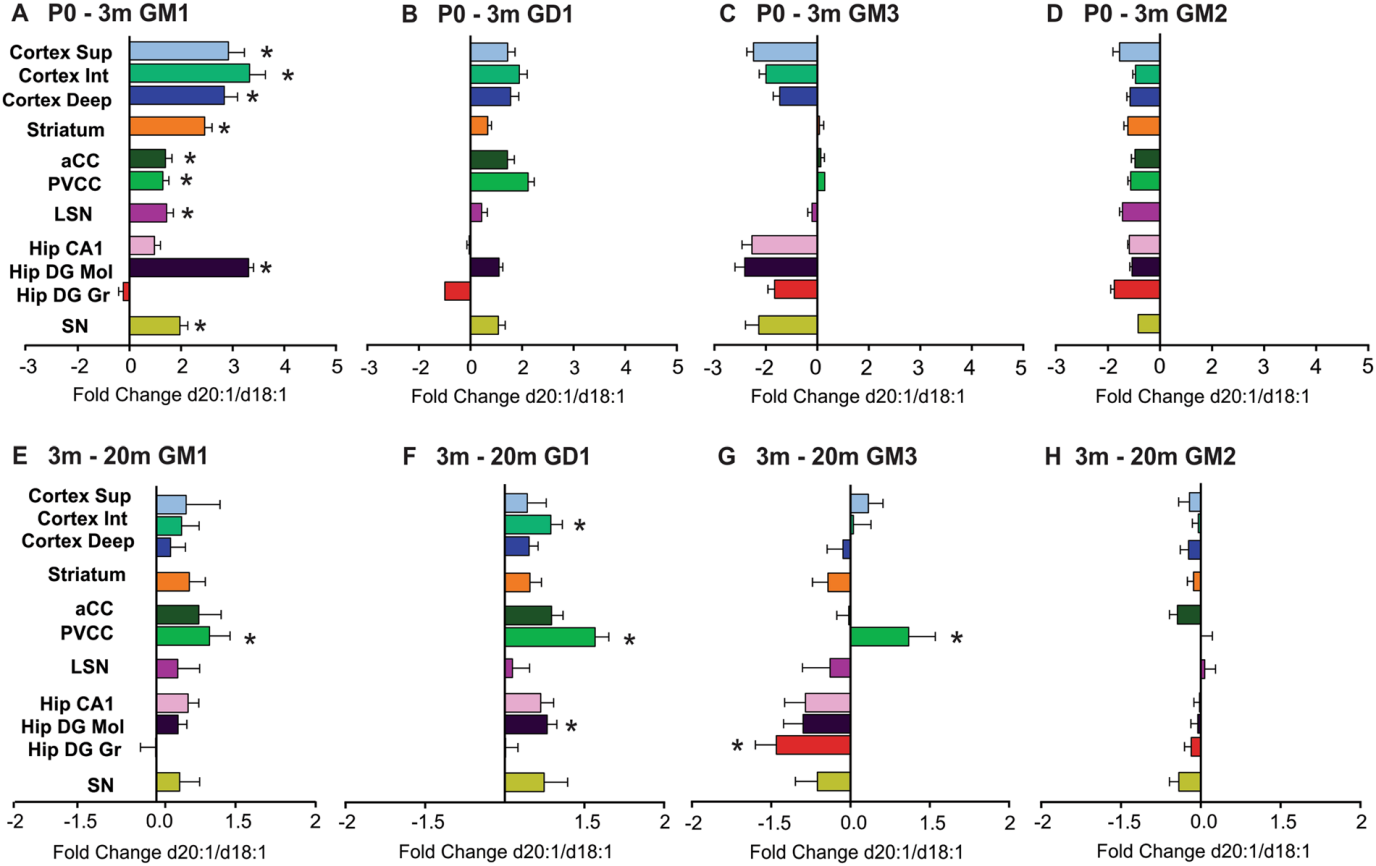
Differential alterations in LCB during early brain development and adulthood in Fisher rats. Overall age effects of d20:1/d18:1 ratio during post-natal rat brain development and adulthood. Quantification of the fold-change in the d20:1/d18:1 ratio between P0 - 3 m (**A–D**) and 3 m–20 m (**E–H**). Ganglioside GM1 shows a significant fold increase between P0 and 3 m (**A**) which only continued to increase in the PVCC during adulthood (**E**). Ganglioside GD1 increased in all brain regions except the CA1 and DG gr layers of the hippocampus in early development (**B**) with a few regions continuing to increase into old age (**F**). Ganglioside GM3 showed either a decreased or stable LCB ratio in early development (**C**) which remained unchanged throughout adulthood except for the PVCC, which increased, and the DG gr, which decreased (**G**). Ganglioside GM2 LCB ratio decreased significantly in early development across the brain (**D**) and remained stable throughout adulthood (**H**). In order to calculate the global changes in early development and adulthood, an average value for the d20:1/d18:1 ratio was calculated (either P0 or 3 m) and used for comparison to each animal in the later time point (3 m or 20 m). Data represented as group Means ± SEM, *indicates statistical significance, p < 0.05, via two-way ANOVA, Tukey multiple comparisons test, n = 10 for each time point.

In order to make global anatomical and age-dependent comparisons between all 11 regions of interest in d20:1/d18:1 content, the fold change was calculated between P0 and 3 m old rats (Fig. 6A–D). This time period corresponds to the early post-natal stages of neurodevelopment and also served as the time period of the greatest observed changes in LCB length in the current study. We also calculated the fold change between 3 m and 20 m, a time period corresponding adolescence and old age in Fisher rats (Fig. 6E–H).

There was a 1.5–4 fold increase in the ratio of d20:1/d18:1 species between P0 and 3 m for ganglioside GM1 in most brain regions examined, with the exception of the DG gr layer of the hippocampus, which remained unchanged. The largest fold increases occurred in the cerebral cortex, striatum, and the DG mol (Fig. 6A). There was a 1.5–3 fold increase in the d20:1/d18:1 ratio for ganglioside GD1 between birth and 3 m, with the exception of the CA1 and DG gr of the hippocampus, in which the former remained unchanged, and the latter decreased 1.5 fold (Fig. 6B). The largest fold increase in GD1 d20:1/d18:1 content during early development occurred in the cerebral cortex and white matter regions. GM3 d20:1/d18:1 content decreased 1.5 to 3 fold across the brain with the exception of the two white matter regions, the LSN, and the striatum which remained unchanged (Fig. 6C). GM2 d20:1/d18:1 content decreased roughly 2 fold across all brain regions examined, with the largest drop occurring in the superficial layer of the cortex and DG gr of the hippocampus (Fig. 6D).

During adulthood, the PVCC showed the largest fold increase for gangliosides GM1, GD1, and GM3 (Fig. 6E–G). The PVCC was the only brain region which the d20:1/d18:1 ratio was significantly altered between 3 and 20 m of age. For ganglioside GD1, the PVCC along with the intermediate layers of the cerebral cortex and DG mol layer of the hippocampus had significantly elevated d20:1/d18:1 ratios, all other regions remained either unchanged or increased less than 50% during adulthood (Fig. 6F). The PVCC was the only region where the LCB of GM3 was significantly increased between 3 and 20 m, however, the DG gr layer of the hippocampus showed a significant decrease within the same period, while all other brain regions remained statistically unchanged (Fig. 6G). There were no significant alterations in the d20:1/d18:1 ratio of GM2 between 3 m and 20 m of age (Fig. 6H).

## Discussion

Work in this study describes the age-dependent changes in the d20:1/d18:1 ratio of gangliosides across multiple cortical, subcortical, and white matter regions of the brain using MALDI IMS. It is the first to examine age-related alterations in the LCB of minor ganglioside components GM2 and GM3 which are currently gaining interest as potential mediators of neurodegeneration in a number of neurodegenerative diseases and injuries^7–9,13,27^. This work builds upon previous studies by Weishaupt *et al*.^24^, which described regional differences in the d18:1/d20:1 ratio of ganglioside GM1 in 3 m old Fisher rats, as well as the work of Sugiura *et al*.^20^, which was the first to use MALDI IMS to described age-dependent changes within hippocampus of mice. The current study confirmed many of the findings from the aforementioned studies pertaining to complex gangliosides GM1 and GD1. For example, Weishaupt *et al*.^24^ used similar regions of interest in the cortex and found, like the current study, that the lowest d20:1/d18:1 ratio occurred in the deep layers of the cortex, with significantly higher ratios in the intermediate and superficial layers. Anatomically, the intermediate and superficial layers of the cortex have a particularly high density of neurons which receive inputs from cholinergic neurons in the basal forebrain^30^ while the deep layers of the cortex have high connectivity to thalamic nuclei^31^. A heterogeneous expression of A-series gangliosides have also been reported in various layers of brain cortices of normal and injured mice. Specifically, Kwak *et al*.^32^ found that ganglioside GM3 was upregulated in deep layers of the cortex (layers 4–6) after an MCAO stroke injury while GM1 was upregulated in more superficial layers (2–3) as well as the deep layers. The regional differences in the d20:1/d18:1 ratio observed in the current study may also reflect important functional differences in connectivity within the cortex that play a role in normal brain aging as well as the brain’s response to injury, however, this connection remains unclear and would be an interesting avenue of further investigation.

We also observed significant regional differences within the various layers of the rat hippocampus from 3 m of age through to 20 m. The DG mol showed the highest ratio of d20:1/d18:1 gangliosides followed by the CA1 and DG gr. Sugiura *et al*.^20^ reported an age-dependent accumulation of d20:1 species of ganglioside GD1 compared to d18:1 species in the DG molecular layer of the mouse hippocampus. While this age-dependent accumulation was also observed in the current study, the CA1 region was only observed to increase between P0 and 12 m of age, while the DG gr showed a decrease in d20:1/d18:1 between P0 and 20 m. This data suggests that not all layers of the hippocampus show an increase in GD1 d20:1/d18:1 ratio between birth and old age, which may be related to the various functions of different anatomical domains within the hippocampus. For example, the DG mol is known to be a critical area of connectivity in the perforant pathway, which receives input from the entorhinal cortex and sends connections to the CA3^33^. It has been previously suggested that the high proportion of d20:1 species observed in the DG mol could be linked to the particular vulnerability of this perforant pathway to neurodegeneration during the development of Alzheimer’s disease^34^. Similarly, the sub-cortical structures examined in the current study are particularly vulnerable to the development of neurodegenerative diseases and perturbations in ganglioside homeostasis, as previously mentioned. Treatments focused on restoring depleted complex ganglioside GM1 have been therapeutically promising both in pre-clinical models of neurodegenerative diseases and clinically^35–37^, pointing to the importance of understanding these ganglioside perturbations during aging. Interestingly, very different patterns of d20:1/d18:1 LCB ratios were observed for simple ganglioside GM3 among these sub-cortical structures throughout the lifespan of the Fisher rats. The LCB ratio dropped during early development only in the SN (similar to the hippocampus and cortex) while levels remained unchanged in the striatum and LSN. This regional and age-dependent heterogeneity in GM3 LCB suggests that the d20:1 species of GM3 is differentially regulated within these subcortical structures and may be an indicator of regional vulnerability to damage and/or disease during aging.

While information on general alterations in the LCB length of simple gangliosides is scarce, our findings do not match with what has been hypothesized to occur based on previous studies conducted on complex gangliosides. Specifically, the age dependent accumulation of d20:1 species in the brain has been suggested to be a hallmark of the aging process^22^, however this assertion was based on the analysis of complex gangliosides only. The results of the current study demonstrated that, relative to the d18:1 species, d20:1 content generally decreased between P0 and 3 m of age for gangliosides GM2 and GM3 and this ratio remains fairly constant throughout adult life. One potential reason for the observed decrease in the d20:1/d18:1 ratio in the early life of the rat may be related to the role of these gangliosides during neurodevelopment. It is possible that the observed decrease in d20:1 content in simple gangliosides may be due to an increase in d18:1 content during the first 3 months, however, this remains unclear in the current study and is thus an important caveat. Future studies which examine and compare alterations in individual ganglioside species may address this pitfall.

Overall, data from this study showed that the greatest changes in LCB composition occurred between P0 and 3 m of age in the rat. This finding is in accordance with previous literature, using alternative techniques, focused on the very early stages of development^19,21^. We demonstrated that the alterations in LCB length between P0 and 3 m of age was highly variable depending not only on the ganglioside species, whether it be simple or complex, but more importantly on the brain region as well. In addition to examining the changes in LCB length in the early stages of rat brain development, we included a detailed report of the LCB length changes in the mature brain in order to determine if there are distinct regions and/or gangliosides that are particularly connected to the process of aging in the rat brain. Interestingly, the PVCC showed the largest fold increase in the d20:1/d18:1 ratio during adulthood for complex gangliosides GM1 and GD1, as well as the pro-apoptotic simple ganglioside GM3. This accumulation of d20:1 species in the PVCC suggests that this region of the brain may be particularly susceptible to the effects of aging and neurodegeneration in rats. Indeed, alterations in gangliosides abundance has been shown to produce abnormalities in white matter^38^ and more recently, Di Pardo, Amico, and Maglione^10^ found decreased levels of complex gangliosides in the corpus callosum of a mouse model of Huntington’s disease. Moreover, the PVCC was the only region that showed an age-dependent increase in the proportion of simple ganglioside GM3 d20:1. This finding in particular supports the notion of susceptibility to neurodegeneration due to the toxic nature of GM3 accumulation reported in the literature^5,6,39,40^, the reported accumulation of GM3 in senescence-accelerated mice^41^, as well as increased simple ganglioside content in the brains of both animal models and clinical patients with AD^12–14^. Regional differences between the two sub-regions of white matter observed in the current study may also have clinical relevance. In a study using magnetic resonance imaging to evaluate white matter hyperintensities in human patients with cerebrovascular diseases, the authors found that PVWM hyperintensities, but not deep white matter hyperintensities, were associated with aging and the incidence of dementia^29^. Future studies should focus on evaluating the mechanism for ganglioside LCB alterations during aging in order to determine whether there are links to white matter-specific pathological changes associated with brain aging and disease.

Although the mechanisms involved in the age-dependent accumulation of d20:1 gangliosides are not fully understood, there are several theories as to what may contribute to this finding. Firstly, it has been suggested that different types of enzymes, or enzyme modulators capable of distinguishing between LCB, may be present and are differentially active throughout neurodevelopment and aging^18,19^. Alternatively, Palestini *et al*.^19^ suggested that d20:1 intermediates may be preferentially used during biosynthesis and recycling of ganglioside degradation products with aging. Rosenberg and Stern 1966 were one of the first to report an age-dependent increase in d20:1 sphingosine in the murine brain. In that study, using whole brain extracts, they also found that there was an age-dependent decrease of stearic acid content in ganglioside fractions. Overall, these studies along with the current study suggest that understanding the role of LCB length in mediating in neurodevelopment and brain aging are worthy pursuits for continued investigation.

MALDI IMS is currently the only technique capable of simultaneously providing accurate detection, ionic abundance, and anatomical distribution data of gangliosides based on both their oligosaccharide and ceramide moieties, making it a powerful analytical tool for the study of gangliosides in the brain. The evolution of MALDI instrumentation along with refined sample preparation protocols allows for very high resolution imaging for the visualization and analysis of discrete anatomical structures within intact tissue sections. This technique, however, is not without drawbacks. It is considered a semi-quantitative technique due to variability in sample preparation and instrumentation between runs. Additionally, detection of multisialylated ganglioside species such as GD1 are susceptible to in-source fragmentation and ionize attached to salt adducts [Na+] and [K+]^42^. These factors dampen the signal obtained for this species and complicate the interpretation of GM1 data as well. However, because the current study quantified the ratio of one species to another within the same spectrum (d20:1/d18:1), direct comparisons between images and individual species abundance was avoided, thus reducing the effects of dampened signal and error produced by between-scan variability. In the end, the numerous benefits of MALDI IMS outweigh the pitfalls and it continues to be the most valuable tool for the investigation of membrane lipids in the brain.

## Conclusion

Overall, data from our study, for the first time, provide insight into the changing composition of simple ganglioside LCB throughout the brain during aging and provide a more in depth examination of LCB alterations in complex gangliosides. This study contains the most detailed examination of the anatomical distribution of d18:1 and d20:1 ganglioside species to date in order to better understand the role of these membrane lipids during aging. Detailed analyses of ganglioside anatomical distribution patterns and abundance during healthy and pathological aging may provide valuable insight for the creation of effective lipid-derived therapeutics for neurodegenerative diseases and injuries.

## Electronic supplementary material

Supplementary Information
