# Supplementary material for: Age-dependent and regional heterogeneity in the long-chain base of A-series gangliosides observed in the rat brain using MALDI Imaging
Source: Sci Rep. 2017 Nov 23;7:16135. doi: 10.1038/s41598-017-16389-z (PMC5701003; doi:10.1038/s41598-017-16389-z)
Supplement: Supplementary file 1 — Supplementary Information [file 41598_2017_16389_MOESM1_ESM.pdf]

Supplemental Information:

Age-dependent and regional heterogeneity in the long-chain base of A-series gangliosides observed in the rat brain using MALDI imaging

Sarah Caughlin[a], Shikhar Maheshwari[a], Nina Weishaupt[a], Ken K-C Yeung[b], David Floyd Cechetto[a] & Shawn Narain Whitehead[a]

[a] Vulnerable Brain Laboratory, Department of Anatomy and Cell Biology, Schulich School of Medicine and Dentistry, University of Western Ontario, London, ON, Canada, N6A 5C1

[b] Department of Chemistry, Department of Biochemistry, Schulich School of Medicine and Dentistry, University of Western Ontario, London, ON, Canada, N6A 5C1

*Corresponding Author:

Shawn N. Whitehead
Dept. Anatomy and Cell Biology, Schulich School of Medicine, Western University , London, ON, Canada N6A 5C1, shawn.whitehead@schulich.uwo.ca

Supplemental Figure 1

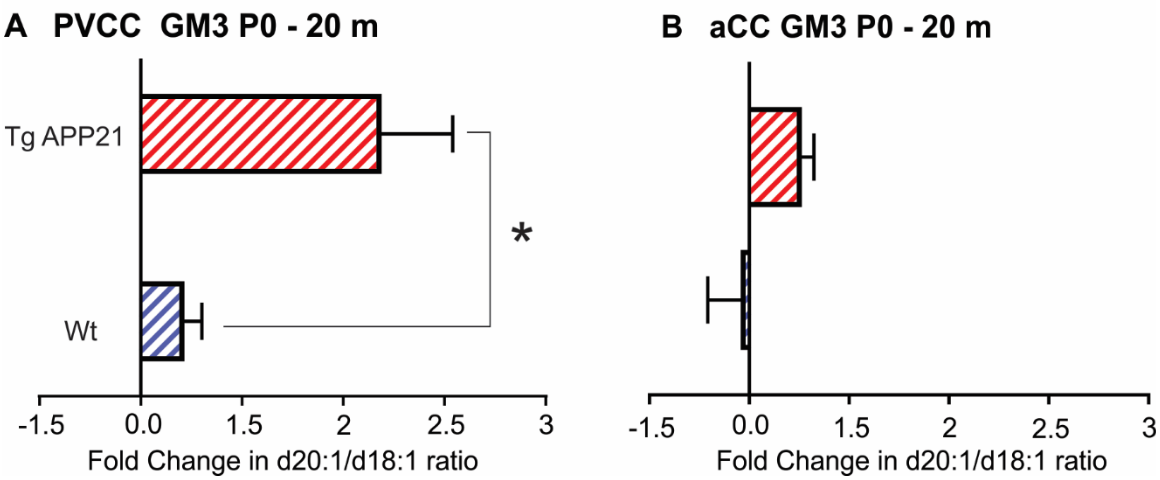

Supplemental Figure 1. Increased d20:1/d18:1 GM3 ratio in PVCC between birth and old age in TgAPP21 rats. Quantification of MALDI IMS data showing the fold change in d20:1/d18:1 signal between birth (P0) and old age (20 m) in the PVCC (A) and corpus callosum (B). Tg APP21 rats showed a significant increase in the d20:1/d18:1 LCB ratio between birth and old age compared to their Wt counterparts in the PVCC. A similar pattern was observed in the aCC, however, the fold change was not statistically different in this region. This transgene difference explains the high degree of variability observed at each individual time point in white matter regions. Data represented as group Means ± SEM,* indicates statistical significance, $p < 0.05$, via student's t-test, n=5 for each time point.

Supplemental Figure 2

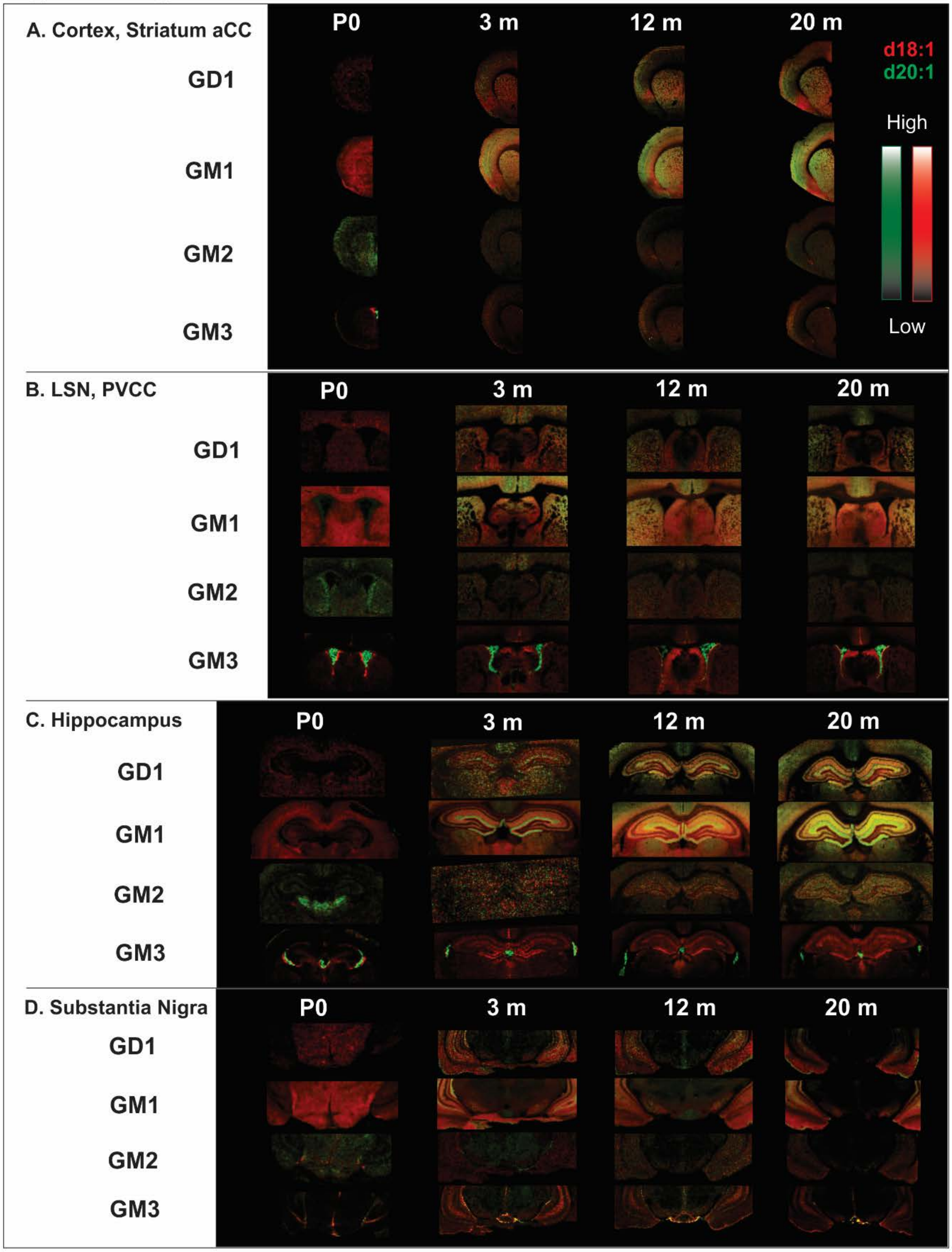

Supplemental Figure 2. MALDI IMS images of A-series gangliosides. MALDI IMS overlay images are presented for each A-series ganglioside species examined in the current study at each of the 4 time points (P0, 3 m, 12 m, 20 m – from left to right). Four tissue sections were used per rat to analyze the 11 regions of interest represented on figures 2-5. D20:1 levels of complex gangliosides were very low in P0 rats, thus the d18:1 species (displayed in red) were predominant. Figures A-D show the distribution of d18:1 (red) and d20:1 (green) species of each A-series ganglioside across the cerebral cortex, striatum, and aCC (A), LSN and PVCC (B), hippocampus (C), and SN (D) at each time point. Images were overlaid and pseudo-coloured using Image J software. No normalization was done to modify the presented images.
